# Real-Time Postural Feedback to Optimize Ergonomics and Musculoskeletal Health in Ophthalmology Residents: A Canadian Pilot Quality Improvement Study

**DOI:** 10.7759/cureus.88736

**Published:** 2025-07-25

**Authors:** Matt Bolis, Anubhav Garg, Brian Chan

**Affiliations:** 1 Faculty of Health Sciences, McMaster University, Hamilton, CAN; 2 Department of Surgery, Division of Ophthalmology, McMaster University, Hamilton, CAN

**Keywords:** clinical ergonomics, medical trainee wellness, musculoskeletal pain, ophthalmology residents, physician well-being, postural biofeedback, posture monitoring, quality improvement, resident education, wearable posture device

## Abstract

Background

Musculoskeletal (MSK) pain is a common occupational concern in ophthalmology, often associated with the sustained and ergonomically demanding positions required during clinical and surgical activities. Tasks such as slit-lamp examinations, indirect ophthalmoscopy, and microscope-assisted procedures may contribute to postural strain. Despite this, ergonomics remains an underemphasized component of resident education, even though physical strain during training can influence long-term clinical performance and physician well-being. This pilot study investigates whether the UPRIGHT GO 2, a wearable posture trainer, can improve posture and reduce MSK pain in ophthalmology residents.

Methodology

This prospective, interventional, proof-of-concept case series recruited five postgraduate year (PGY) 2 to 5 ophthalmology residents at McMaster University. Each participant wore the UPRIGHT GO 2 device over the following four distinct two-week phases: baseline, training, short-term testing, and long-term testing. During the baseline phase, the vibration mode was turned off to establish baseline posture data. In the training phase, the vibration mode was activated with a 30-second delay to provide real-time posture feedback. The short-term testing phase was conducted with the vibration turned off to assess short-term retention effects. The long-term testing phase, also with vibration off, was performed six weeks after short-term testing to evaluate long-term effects. All participants attended a brief standardized educational session before device use, which reviewed ergonomic principles relevant to ophthalmology, including optimal posture during slit-lamp examinations, indirect ophthalmoscopy, and microscope-guided procedures. The primary outcome was the percent time spent in an upright posture. The secondary outcome was MSK pain, assessed using a modified Nordic musculoskeletal and numerical pain rating scale.

Results

All participants (N = 5, 100%) completed the baseline and training phases, with two residents (n = 2, 40%) completing the full study through long-term testing. The mean proportion of time spent in an upright posture increased from 68.9% at baseline to 78.5% during the training phase, coinciding with the activation of vibration-based feedback. This improvement declined to 66.8% during short-term testing and further to 52.4% at long-term follow-up (n = 2, 40%), suggesting a potential attenuation of effect in the absence of continued reinforcement. MSK pain scores followed a similar pattern: mean scores increased slightly from 6.6 to 7.4 post-baseline, then declined post-training (6.4), post-short-term testing (6.5), and reached their lowest average at long-term follow-up (3.5, n = 2, 40%). All participants demonstrated either stable or improved pain scores, with two residents exhibiting concordant improvements in both posture and pain. These findings suggest that wearable feedback devices may enhance ergonomics and mitigate MSK symptoms among ophthalmology residents when incorporated into clinical training environments.

Conclusions

The UPRIGHT GO 2, combined with an educational intervention, may provide short-term ergonomic benefit for posture and MSK pain in ophthalmology residents. However, long-term posture retention varied. Limitations include the small sample size and device data fidelity. Larger studies are needed to validate these findings and guide ergonomic strategies in medical training.

## Introduction

The well-being of medical learners has become an increasingly emphasized topic. Well-being is a multidimensional concept that encompasses intellectual, occupational, social, mental, and physical domains [1]. However, a recent scoping review found a scarcity of interventions focusing on the well-being of medical learners in Canada [2]. In the field of ophthalmology, musculoskeletal (MSK) pain is a frequently reported occupational concern, often associated with ergonomically demanding clinical activities, such as indirect ophthalmoscopy, slit-lamp examinations, and microscope-guided procedures [3,4].

A national Canadian survey revealed that 50% of ophthalmologists and residents experienced MSK pain, which was most often attributed to repetitive tasks and working in ergonomically suboptimal positions [5]. Similar findings have been reported globally, with studies showing high rates of work-related MSK pain among ophthalmologists [6-8]. For instance, 78% of ophthalmologists reported low back pain and 41% reported neck pain, with symptoms particularly prominent during slit-lamp examinations and surgical procedures [9]. These physical stressors, if left unaddressed during training, may contribute to long-term health complications and affect clinical performance and career longevity [10].

Despite its relevance, ergonomics remains an underrepresented focus in resident education. Poor posture has also been associated with intervertebral disc degeneration, vertebral misalignment, and nerve compression syndromes that lead to MSK symptoms [11]. Structured ergonomic interventions are rare, and few studies have explored practical, low-barrier solutions within training environments. One such intervention is the UPRIGHT GO 2 device. This compact wearable posture trainer adheres to the upper back and provides vibration-based feedback when a user deviates from a calibrated, upright posture [4,12].

A recent U.S. pilot study using the UPRIGHT GO 2 in ophthalmology residents showed that the device may improve posture during surgical procedures. However, this study did not assess long-term effects or evaluate MSK pain outcomes [4]. It also focused solely on intraoperative posture, without addressing clinic-based ergonomics, despite the routine use of MSK-intensive techniques in outpatient ophthalmology. Moreover, no similar study has been conducted in a Canadian residency program to date. This pilot study aims to address these gaps by evaluating both posture and MSK pain outcomes during clinical and surgical duties in Canadian ophthalmology residents using the UPRIGHT GO 2 device [12]. We hypothesized that real-time posture feedback would reduce MSK pain and promote long-term ergonomic wellness among ophthalmology residents by reinforcing optimal spinal alignment during clinical and surgical tasks.

## Materials and methods

Study design and participants

This prospective, interventional, proof-of-concept case series was conducted at McMaster University in Hamilton, Ontario. Five ophthalmology residents in postgraduate years (PGYs) 2 to 5 were enrolled from the institution’s residency program. PGY1 residents were excluded due to their limited ophthalmology-specific clinical exposure. Participants were recruited voluntarily through direct communication with study investigators, and they provided verbal consent to participate.

As part of the intervention, participants attended a brief educational lecture at the beginning of the study covering optimal posture techniques in ophthalmologic settings (e.g., slit-lamp examination, indirect ophthalmoscopy, microscope use, surgical loupes). They were also sent an educational PowerPoint via email for asynchronous review. The lecture content and accompanying PowerPoint presentation were developed by the investigators specifically for this study.

Each participant underwent height measurement, completed an initial MSK pain survey, and set up their UPRIGHT GO 2 device using the companion smartphone application [12]. Participants were guided through device calibration using the UPRIGHT GO 2 mobile application, which involved sitting in a neutral, ergonomically upright position to establish a personalized reference posture. Participants recorded whether each workday was spent in the clinic or the operating room.

Intervention and study phases

Participants then used the UPRIGHT GO 2 wearable posture training device over four consecutive two-week phases [12]: Baseline (weeks 1-2): The device was worn with vibration feedback disabled to collect initial posture data. Training (weeks 3-4): Vibration feedback was enabled with a 30-second delay to provide haptic cues for postural correction. Short-term testing (weeks 5-6): Vibration feedback was again disabled to assess posture retention immediately following training. Long-term testing (weeks 12-13): Conducted six weeks after short-term testing with vibration off to evaluate sustained ergonomic effects.

Data collection

The primary outcome was the proportion of time spent in an upright posture during clinical activities, expressed as a percentage, as recorded by the UPRIGHT GO 2. The device integrates an accelerometer and gyroscope to detect deviations from a calibrated “upright” position, defined as head and upper trunk aligned at 0° relative to the spine [12]. Posture data were synced to a mobile application and uploaded to REDCap for analysis.

The secondary outcome was MSK pain, assessed using a modified version of the Nordic musculoskeletal questionnaire combined with a numerical pain rating scale (maximum score = 90) [13,14]. The Nordic musculoskeletal questionnaire is a validated survey tool for self-reporting the presence and location of MSK symptoms across nine anatomical regions (neck, shoulders, upper back, elbows, wrists/hands, lower back, hips/thighs, knees, and ankles/feet) over specified time frames [13]. The numerical pain rating scale is a subjective self-report measure in which participants rate their pain severity on a scale from 0 (no pain) to 10 (worst possible pain) [14]. The Nordic musculoskeletal questionnaire is licensed under the Creative Commons Attribution-ShareAlike 4.0 International License [15,16]. The numerical rating scale is licensed under the Creative Commons Attribution 3.0 Unported License [17,18]. Baseline surveys captured reports of pain experienced in the preceding 12 months across the nine specified anatomical regions [13,15]. Follow-up surveys at the end of each phase evaluated pain severity over the prior two weeks using a 0-10 scale for each body region [14,17]. Participants also logged whether each workday was spent in the clinic or the operating room.

Data analysis

Descriptive statistics (mean, standard deviation) were used to summarize demographic data, posture metrics, and pain scores. Paired t-tests compared posture and pain outcomes across study phases relative to baseline. Due to the small sample size and participant attrition in later phases, comparisons at each time point were conducted using complete case analysis, including only those with available data for each specific phase. Subgroup analyses stratified results by work setting (clinic vs. operating room). Between-group comparisons of clinic and surgical environments within each phase were assessed using unpaired t-tests. Given the small sample size, the statistical power of paired t-tests is limited. These findings should, therefore, be interpreted as exploratory, and future studies with larger cohorts are needed to validate these preliminary observations.

## Results

Participant characteristics

Participant demographic characteristics are summarized in Table 1. The table provides an overview of each participant’s PGY level and height at baseline, which is relevant to interpreting ergonomic posture data.

**Table 1:** Demographic characteristics of ophthalmology resident participants.

Resident	Postgraduate year	Height (cm)
A	5	179.1
B	2	160.0
C	4	174.0
D	3	186.7
E	5	163.8

Postural outcomes

All five participants (N = 5, 100%) completed the baseline and training phases. Two participants (n = 2, 40%; Residents B and C) completed the short-term and long-term testing phases. Across the cohort (N = 5, 100%), the average proportion of time spent in an upright posture increased from 68.9% at baseline to 78.5% during the training phase, reflecting improvement during active vibration feedback. During short-term testing (vibration off), the mean upright time declined to 66.8% (n = 2, 40%), and further to 52.4% during long-term testing six weeks later (n = 2, 40%), suggesting a possible attenuation of effect over time in the absence of active reinforcement.

**Figure 1:**
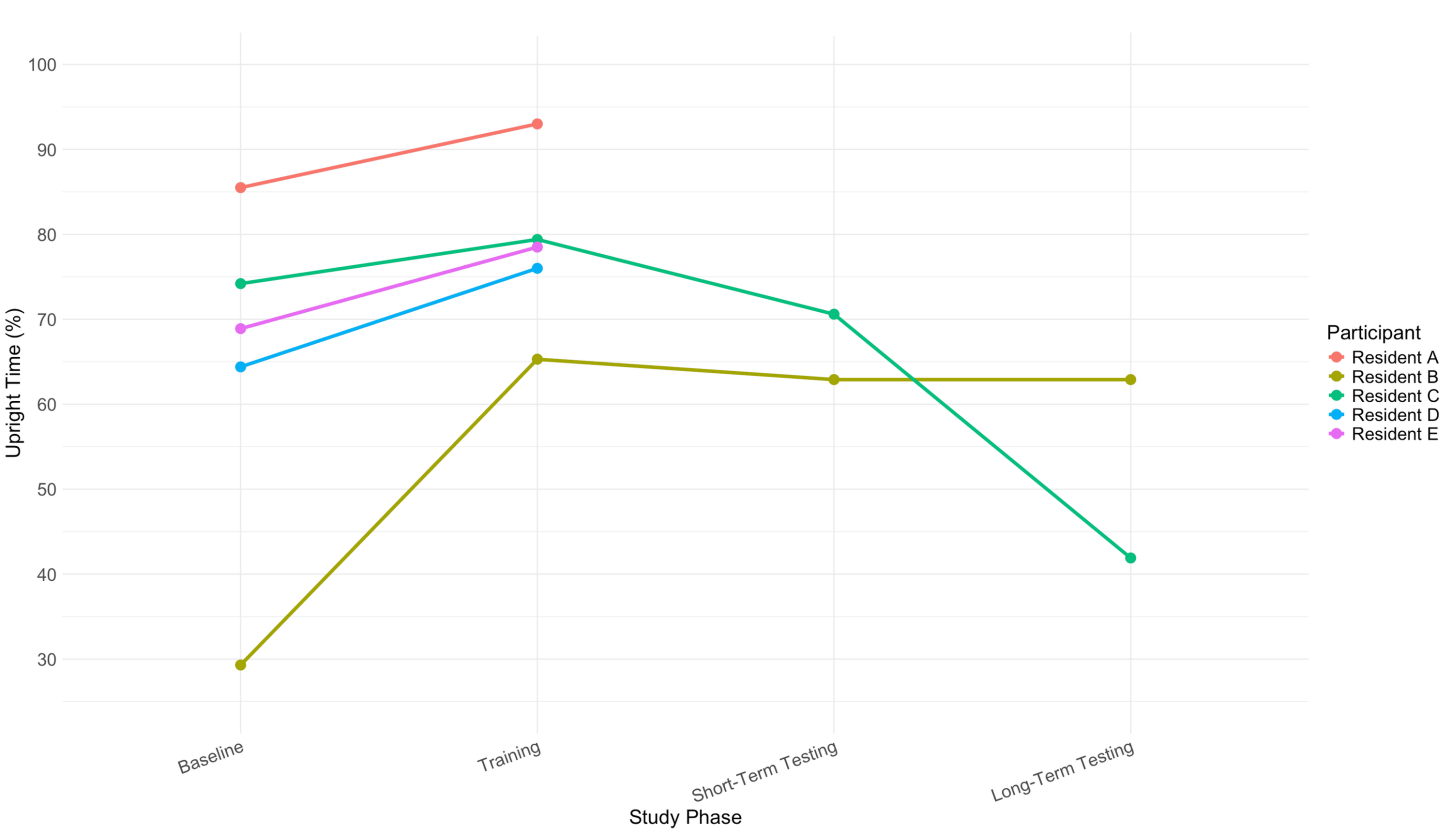
Upright posture time across study phases by participant. Line graph illustrating changes in upright posture time (%) among ophthalmology residents (A-E) across the four study phases: baseline, training, short-term testing, and long-term testing. Values represent the proportion of time each participant maintained an upright posture.

Postural trends across all residents are summarized in Figure 1, which depicts the proportion of time spent in an upright posture across each study phase. Individual-level data demonstrated variability in both baseline posture and magnitude of improvement. Resident A showed improvement from 85.5% at baseline to 93.0% during training. Resident B demonstrated substantial improvement from 29.3% to 65.3%, with minimal decay during short-term testing (62.9%). Resident C increased from 74.2% at baseline to 79.4% during training, followed by a decline to 70.6% in short-term testing and 41.9% in long-term testing. Resident D improved from 64.4% at baseline to 76.0% during training. Resident E similarly increased upright posture from 68.9% (baseline) to 78.5% (training).

Pain outcomes

MSK pain scores were evaluated using a modified Nordic musculoskeletal questionnaire combined with a numerical rating scale (maximum score = 90). The mean score at the initial assessment (N = 5, 100%) was 6.6, which increased slightly post-baseline (7.4), and then decreased post-training (6.4). Among the two participants who completed all phases (n = 2, 40%), scores continued to decline post-short-term testing (6.5) and post-long-term testing (3.5). This trend suggests a potential progressive benefit in pain reduction over time, possibly reflecting delayed ergonomic adaptation.

**Figure 2:**
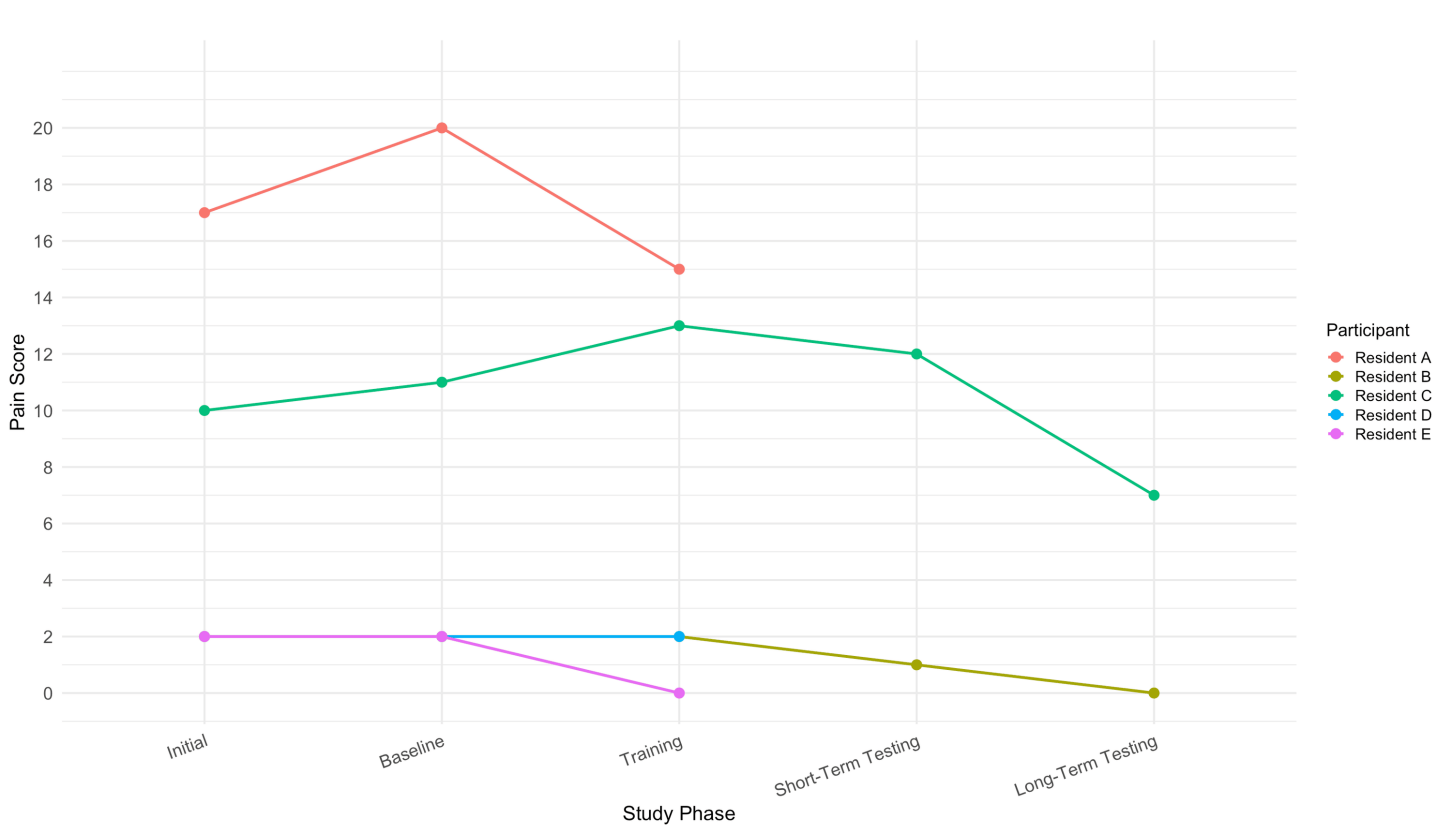
Individual pain scores across study phases. Line graph depicting pain scores reported by individual participants (residents A-E) across the five study phases: initial, baseline, training, short-term testing, and long-term testing. Decreases in pain scores suggest a potential benefit of the intervention over time.

Trends in MSK pain scores over the course of the study are depicted in Figure 2, with scores evaluated at baseline and after each study phase. All participants (N = 5, 100%) demonstrated stable or improved pain scores across their respective study phases. Among those who completed up to the training phase (n = 3, 60%), Resident A improved from a baseline of 20 to 15, Resident E improved from 2 to 0, and Resident D remained unchanged at 2. Of the two participants who completed all four phases (n = 2, 40%), Resident B’s pain decreased steadily from an initial score of 2 to 0 at long-term follow-up, while Resident C’s pain improved from a peak of 13 during training to 7 at long-term testing. Notably, Resident B demonstrated concordant improvements in both posture and pain, whereas Resident C experienced pain improvement despite declining upright posture percentages at long-term follow-up.

## Discussion

This pilot study is the first of its kind in Canada to evaluate the use of a wearable posture-training device, the UPRIGHT GO 2, among ophthalmology residents [12]. Our findings support the feasibility of this approach and highlight both ergonomic benefits and positive outcomes for resident well-being. Among all five participants (N = 5, 100%), the mean upright posture improved from 68.9% at baseline to 78.5% during the training phase, demonstrating that real-time haptic feedback can effectively modify postural behavior. The attenuation of this effect during short-term and long-term testing, where vibration feedback was deliberately deactivated, suggests that reinforcement mechanisms, such as continuous sensory cues, are crucial to sustaining ergonomic habits beyond an initial training period.

Notably, among the two residents who completed all phases (n = 2, 40%), one maintained an improved posture (Resident B), while the other (Resident C) experienced a decline at long-term testing. Despite this decline, pain scores continued to improve, indicating that even intermittent posture improvements can provide symptomatic relief over time. This aligns with evidence from studies involving wearable posture trainers in surgical trainees, which have reported improved ergonomics and reduced MSK strain [19,20].

Pain outcomes followed a similar trajectory. Scores increased modestly post‑baseline, possibly reflecting initial ergonomic strain, then declined post‑training and reached their lowest levels at long‑term follow‑up (mean = 3.5/90, n = 2, 40%). All five residents (N = 5, 100%) reported stable or improved symptoms: Resident A improved during training, Resident D remained stable, and Residents B, C, and E experienced sustained reductions through long‑term testing. This suggests that even pilot-level interventions can have meaningful impacts on resident well-being, especially when integrated early in training and supported with reinforcement.

Implications for residency education and wellness

The high ergonomic burden in ophthalmology is well documented, with 46% of Canadian ophthalmologists reporting neck pain, followed by lower back pain (36%) and shoulder pain (28%) within the past year. Nearly half (48.3%) attributed these symptoms directly to operating room activities, which often involve repetitive motions, awkward postures, and sustained neck torsion during procedures [5]. Despite this, formal ergonomic education remains limited in most residency curricula [21]. This study uses a quality improvement approach by integrating wearable technology into an existing educational framework, offering residents both posture-related self-monitoring and real-time feedback. This combined strategy promotes sustainable wellness habits and aligns with broader efforts to foster resilience in medical training.

Trauma from MSK strain often accumulates gradually. Early interventions such as posture training could mitigate not only physical symptoms but also protect against career attrition. In this sense, the UPRIGHT GO 2 device served as a practical addition to resident education, an inexpensive, user-controlled tool that promotes self-awareness and aligns with residency wellness goals [12].

Study limitations and future directions

While the study’s small size and single-site design limit generalizability, the positive trends observed justify larger, multi-center feasibility trials. Additionally, while paired comparisons were made, the study was not powered to detect statistically significant changes, an accepted limitation in such early-phase pilot studies. Future studies may explore the impact of adding intermittent or ongoing vibration reinforcement, embedding training into the formal curriculum, and examining outcomes in diverse clinical settings. Use of mixed methods, combining quantitative tracking with qualitative interviews, could reveal barriers to implementation and inform future ergonomic training protocols. A controlled trial comparing standard ergonomic instruction versus wearable biofeedback may yield stronger evidence for integration into accreditation standards. Lastly, the study evaluated only one type of posture training device (UPRIGHT GO 2). Future investigations could explore the comparative effectiveness of alternative ergonomic technologies.

## Conclusions

This pilot study, the first of its kind in Canada, demonstrates that wearable posture training technology can be effectively integrated into ophthalmology residency education. Participants did not report engagement in other structured lifestyle interventions during the study period, helping isolate the effects of the intervention. Improvements in ergonomic behavior and MSK symptoms observed among participants highlight the potential of such tools to support the physical well-being of trainees. In addition to wearable technology, the preliminary educational intervention appeared to aid in early behavior change, suggesting that combining structured ergonomics education with real-time feedback may offer a sustainable approach. While two participants completed all four study phases, the consistent trends observed support further investigation in larger samples. Given the high ergonomic demands of ophthalmic practice, early incorporation of posture training may contribute to healthier, longer careers and more consistent delivery of care. These findings support continued exploration of wearable technology as a practical and scalable addition to residency curricula, reinforcing the importance of prioritizing provider health as a foundation for high-quality patient care.
